# Developing a checklist for research proposals to help describe health service interventions in UK research programmes: a mixed methods study

**DOI:** 10.1186/1478-4505-12-12

**Published:** 2014-03-04

**Authors:** Hannah Dorling, Donna White, Sheila Turner, Kevin Campbell, Tara Lamont

**Affiliations:** 1 National Institute for Health Research Evaluation, Trials and Studies Coordinating Centre (NETSCC), University of Southampton, Alpha House, Enterprise Road, Southampton SO16 7NS, UK

## Abstract

**Background:**

One of the most common reasons for rejecting research proposals in the National Institute for Health Research (NIHR) Health Services and Delivery Research (HS&DR) Programme is the failure to adequately specify the intervention or context in research proposals. Examples of failed research proposals include projects to assess integrated care models, use of generic caseworkers, or new specialist nurse services. These are all important service developments which need evaluation, but the lack of clarity about the intervention and context prevented these research proposals from obtaining funding. The purpose of the research presented herein was to develop a checklist, with key service intervention and contextual features, for use by applicants to the NIHR HS&DR Programme to potentially enhance the quality of research proposals.

**Methods:**

The study used mixed methods to identify the need for and develop and test a checklist. Firstly, this included assessing existing checklists in peer-reviewed literature relevant to organisational health research. Building on existing work, a new checklist was piloted. Two reviewers used a small sample (n = 16) of research proposals to independently assess the relevance of the checklist to the proposal and the degree of overlap or gaps between the constructs. The next two stages externally validated the revised checklist by collecting qualitative feedback from researchers and experts in the field.

**Results:**

The initial checklist was developed from existing checklists which included domains of intervention and context. The constructs and background to each were developed through review of existing literature. Eight researchers provided feedback on the checklist, which was generally positive. This iterative process resulted in changes to the checklist, collapsing two constructs and providing more prompts for others; the final checklist includes six constructs.

**Conclusions:**

Features relating to intervention and context should be well described to increase the quality of research proposals and enhance the chances of the research receiving funding. Existing checklists do not have enough focus on areas relevant to research proposals in complex health service interventions, such as workforce. A formative checklist has been developed, and tested by end users. Tentative findings suggest usefulness and acceptability of such a tool but further work is needed for full validation.

## Background

The National Institute for Health Research (NIHR) Health Services and Delivery Research (HS&DR) Programme funds research in the UK for managers and service leaders on the quality, access, and organisation of health services, including evaluations of how the National Health Service (NHS) might improve delivery of services. Additionally, the programme aims to support research that is likely to lead to changes in practice that will have a significant impact, is likely to generate new knowledge of direct relevance to the NHS, and has the potential for findings to be applied to other conditions or situations outside the immediate area of research [1]. When considering research proposals in a competitive funding process, weight is given to the team’s track record and the appropriateness of the research design. However, one of the most common reasons for which the funding board rejects research proposals is failure to adequately specify the intervention or contextual features in research proposals. Many complex interventions are staff-based, but little information is given on the composition of the team in terms of skill-mix and grade-mix. Other factors which may be harder to measure include aspects of organisational culture, including levels of staff engagement or the degree to which the organisation is data-driven or embraces change. Examples of failed research proposals include projects to assess new integrated care models, use of generic caseworkers, specialist nurse service, and streamlined services. These are all important complex service developments that need evaluation, but have lacked clarity about the intervention and context, which meant that opportunities to obtain funding for valuable research were missed.

Complex interventions are often described as “interventions that contain several interacting components, but they have other characteristics that evaluators should take into account” [2]. The Medical Research Council’s complex interventions guide notes that the ‘active ingredient’ in healthcare interventions can be challenging to describe [3]. This can include components of an intervention such as behaviours and methods of organising and delivering those behaviours, for example different types of practitioners, settings, and locations. This is unlike ‘simple’ interventions, such as a drug where researchers face fewer problems in providing details where, for instance, the intervention can be defined in terms of dose and frequency [4], whereas one of the key details when evaluating complex interventions is to consider whether they are generalizable to everyday practice. Therefore, it is essential to understand the whole range of potential effects and how they may alter, for example, among recipients, staff, or between sites [2]. Such initiatives may be poorly described, such as exact staff and grade mix which limits the fidelity and reproducibility of the intervention. Many workforce type interventions build upon existing services and it may be difficult to isolate the particular features of the intervention which are new and distinct from ‘care as usual’, as required for experimental or quasi-experimental design. Other factors which may be harder to communicate include aspects of organisational culture, including levels of staff engagement or the degree to which the organisation is data-driven or embraces change.

Often in health services research there is a blurring between intervention and context [5], particularly in workforce interventions, which can increase the difficulty of designing robust evaluations. A lack of clarity could in part be due to the complex nature of contexts; therefore, the role of context in intervention development needs to be well understood to reduce ambiguity [6]. This blurring between intervention and context may make it more challenging when writing research proposals, due to the multifaceted nature of such interventions. For instance, important elements of the research proposal may be overlooked or undervalued, but need to be embedded within the research. A checklist may be useful to help researchers identify these elements and help explicitly explain how they will be considered in a research proposal.

A checklist can be a useful prompt for completeness of key information or processes in any given activity [7]. For example, Taylor proposes a checklist with classified features of context which may be particularly relevant when evaluating patient safety interventions [6]. Other tools typically focus on checklists that aim to improve the reporting of research, such as the CONSORT statement for clinical trials [8] and SQUIRE guidelines on implementing service improvements [9]. Similarly, other publications have presented evidence, systematically reviewing studies that evaluate interventions [10]. For instance, checklists have been used to ascertain whether elements of the intervention were fully described. Glasziou et al. reviewed 80 studies and concluded that over half were missing details [11]. Schroter et al. developed a checklist and used it to evaluate 51 trials published in the *British Medical Journal*[12]. More recently, Douet et al. used this checklist to assess whether NIHR Health Technology Assessment randomised controlled trials published in the *Health Technology Assessment* journal were described in sufficient detail to replicate in practice [13]. Much of this work is focused on research around therapeutic or diagnostic interventions or procedures, which tend to be more defined and well-bound than complex people-based service interventions. Nevertheless, all the checklists have a common theoretical underpinning; they are a mnemonic device, with a list of components or constructs in order to perform a certain task. This function makes them useful in evaluation, since a systematic approach can be used to determine value complex factors. However, none of these checklists focus on improving the quality of research proposals, but their content and benefits are well recognised and valuable to researchers.

Taking these points into consideration, it was felt that the development of a checklist may be a useful tool that could enable less experienced applicants to more successfully address the complex set of requirements involved in writing research proposals to the NIHR HS&DR Programme. In turn, it is hoped that such a tool may enhance the quality of research proposals, so the programme can fund important complex service developments which need evaluation. This is not expected to solely answer the problem, but begin to improve the situation. Therefore, the purpose of our study was to develop a checklist, with key service intervention and contextual features [14].

## Methods

A mixed methods approach was used in four stages to meet the aims of the research.

### Stage one

Firstly, an initial checklist was designed by HD and TL, based on a review of existing checklists, reporting standards, and relevant literature. A snowball sampling technique was used to identify literature, which was prompted by reading a patient safety checklist paper [6]. This technique allowed us to produce a collection of relevant articles built around the initial article and facilitated insights into a broad background of research. Literature that was considered was firstly informed by what is needed in research proposals and is relevant to applicants to the programme and, secondly, by an understanding of complex interventions that are delivered by the NHS and their context.

### Stage two

The second stage was to pilot the checklist, by conducting a retrospective review of a sample of previous applications. To provide focus, this sample was kept to research proposals received in response to two NIHR HS&DR invitations to apply for research funding. Since the checklist concentrates on research employing an intervention, secondary, theoretical, and methodological studies were excluded. From the two invitations for funding, 16 full research proposals were included, which covered a balance of service and workforce interventions across a range of health settings and care groups. These research proposals described studies with a wide range of settings and interventions such as training packages, community healthcare services, discharge packages, and hospital design.

Initially, two reviewers (HD and DW) examined 4 out of the 16 research proposals independently, assessing the relevance of the checklist to the proposal, and the degree of overlap between the constructs. The results of this initial investigation were then discussed in person to assess the level of agreement between reviewers. The remaining research proposals were then reviewed and discussed. The two reviewers examined the relevance of the initial checklist to the research proposals and made judgements on whether the checklist constructs were useful, whether the appropriate terminology was used, and whether any elements were missing. Disagreements were resolved through consensus discussion supervised by TL.

### Stage three

The next stage was to validate the content of the checklist and determine whether it was a suitable tool for researchers to use. This was done in several steps. The first step was to gain feedback from researchers who were preparing to submit detailed full proposals to the NIHR HS&DR Programme in response to the invitation to apply for funding for studies on ‘the organisation and delivery of 24/7 healthcare’. Out of the five researchers contacted, three were able to give feedback.

### Stage four

To further validate the checklist, the next stage was to contact researchers who currently had a NIHR HS&DR funded project. A meeting welcoming those working on newly funded projects to the NIHR was used as an opportunity to make contact with researchers. Researchers from each project were asked to complete the checklist for their study (unless they felt their study did not have an ‘intervention’, as the checklist would not be applicable to them). Five researchers were able to give feedback, which was collected by HD via telephone and email, using semi-structured questions. We asked about their experience of using the checklist, its usability, and the suitability of the content of each checklist item. Lastly, the checklist content was assessed by consultant advisors, who provide professional advice to the NIHR HS&DR Programme and regularly view research proposals submitted to the Programme.

## Results

### Stage one

**Figure 1:**
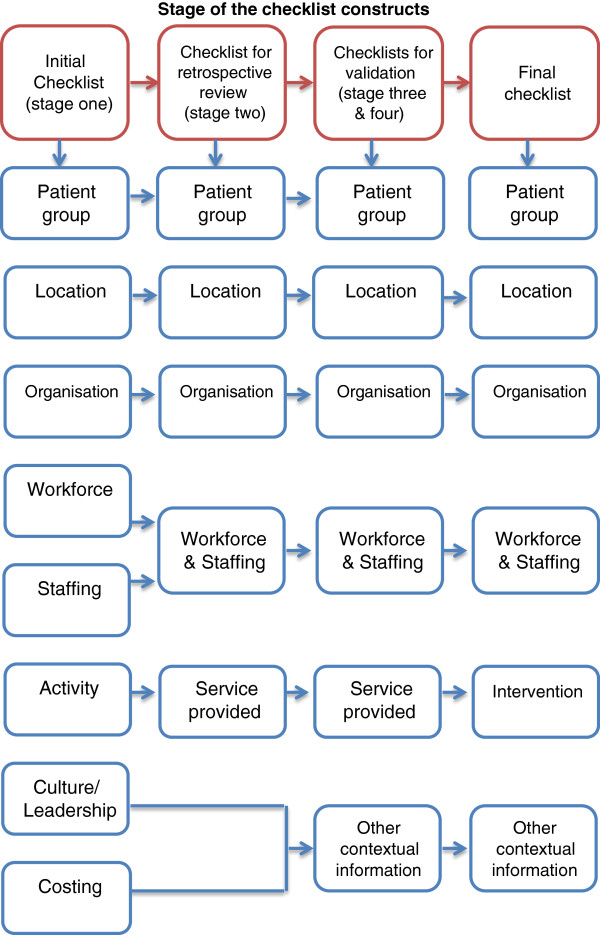
The development of the checklist constructs.

Eight key constructs were identified from the literature review – organisation, location, patient group, workforce, staffing, activity, culture/leadership, and costing (Figure 1). An initial checklist was designed, containing a very brief description of each of these constructs and specific prompts for each. Many of the checklist constructs originated from other checklists, such as those developed in the research by Taylor [4] and Schroter [10], reporting tools [6,7], and other literature on complex interventions [15,16] and context [17,18], including important features such as leadership [19,20] and culture [21].

### Stage two

The checklist constructs were adapted for use by researchers by giving further explanation to clarify the background to each one by considering their relevance to NIHR HS&DR research proposals. Once this had been completed a number of changes were made, which are discussed further on.

### Stages three and four

On the whole, the researchers responded positively to the checklist; a sample of the feedback from researchers is presented in Table 1. The researchers appreciated having a tool to aid them in writing their research proposals and commented that it made them think about factors that they would not have considered previously. Overall, the essence of the checklist appeared to be clearly understood.

**Table 1:** Perceptions of the checklist

**Positive aspects**	“It gives a good visual cue”
	“It gives you the confidence to write a good proposal as it is a good starting point”
	“The checklist reinforced the importance of the big picture rather than a selective focus”
	“It is a very useful tool and has a good outline and structure”
	“The checklist enables you to be more explicit about what to write in research proposals and it clarifies the question”
	“The main question summarises the important core to our research proposals”
	“It is very applicable to the current proposal currently being worked on, especially as there is lots of patients and workforce information”
	“I would certainly read over to match this with the research proposals… and would download it off the website”
	“We used it in our meeting to write the full proposal…the team really liked it!”
	“The checklist was helpful, particularly in thinking through the many and varied aspects of context”
	“Depending on the proposal content, for instance if it was an intervention study, I would access and use it”
	“The most useful box was the contextual information, not many of those details would have been added if the box was not there”
**Concerns and suggestions for improvement**	“A weighting might be useful”
	“Another box which could be added is data sources, for instance who collected it, is there anything unique and the measurement of the outcomes”
	“Maybe it should be used at the initial outline proposal stages?”
	“To improve the checklist need to include points on which other groups are likely to be affected by the change in service delivery”
	“If the language used was more generic i.e., not focussed towards intervention studies it could be more widely employed”
	“The checklist should fit on one page, anything bigger than that would probably put people off from reading it”
	“It would take too long to fill out”

The main construct which researchers had not considered when writing research proposals was the ‘other contextual information’. This may be due to its complex nature, unlike a simple intervention. Some of the principal investigators for service evaluations were clinicians without experience in carrying out complex mixed-methods evaluations. The inclusion of this general construct appeared helpful in prompting researchers to consider issues such as sampling strategy (“how ‘typical’ is my organisation?”) and attribution (“how will I know that the results are not due to the charismatic medical director in that hospital?”) in the research design.

Some researchers also had concerns about the checklist and recommendations for improvements were given. For instance a couple of the researchers interpreted the meaning of a few words differently to what had been intended, therefore, the wording was changed to improve clarity. Other recommendations were made, such as giving a weighting to each category, but this was not applicable to meet the aims the checklist, therefore, no further changes were made.

Consultant advisors from the NIHR HS&DR Programme fed back that some of the wording needed to be updated due a recent reorganisation within the UK’s NHS, but otherwise they felt the checklist was suitable for researchers to use to make a good quality research proposal.

The final checklist includes six constructs, each with a few bullet points to the background (Table 2). Decisions about whether to include or exclude each construct were made during the different stages of the project, as illustrated in Figure 1.

**Table 2:** The final checklist

**Organisation**	Is it clear which organisation(s) are involved in the study?
	Has the proposal considered:
	- How many study sites? How do these differ?
	- How big is the organisation?
	- What type of trust or authority is involved? e.g., Acute trust, ambulance trust, CCG, local authority, care trusts, mental health trusts, etc.
	- Other information on organisational type, e.g., foundation trust status, teaching/research beacon site, CQC or other ratings if relevant
**Location**	Is it clear where the organisation is located?
	Has the proposal considered:
	- What type of area it is, e.g., urban, rural, deprivation, etc.
	- What are the population demographics?
**Patient group**	Is it clear what the case mix of patients is?
	Has the proposal considered:
	- Who is receiving the intervention?
	- How many patients are being seen?
	- The characteristics of the patients, e.g., age group, health status, disease area.
**Workforce and Staffing**	Is it clear who are the main actors involved in service delivery?
	Has the proposal considered:
	- Is the total workforce clearly described? e.g., are the skill, grade, and profession mix stated?
	- How has the staff been selected? Are they already working at the organisation or are they new to the organisation?
	- What if any training will be needed for staff to deploy the intervention?
**Intervention**	Is there enough information about the service or intervention under exploration/examination?
	Has the proposal considered:
	- How is the intervention different from usual care?
	- What does it consist of? What does it ‘look’ like?
	- Is it clear when the intervention started and finished?
	- If there are information materials are these adequately described?
	- If there is a training programme, are there clear details?
**Other important contextual information**	Are there any other contextual aspects which may affect the outcomes of this research and generalizability to other sites?
	For instance, are there particular:
	- Policy initiatives related to the intervention (such as national or regional patient safety or improvement initiatives)
	- Particular issues at the study sites around exceptional leadership, for instance championing by nurse director or clinical team
	- Local features such as unusual geographical or service configuration
	- Events at the site, such as a critical safety incident or top team re-shuffle

### Organisation

Organisation is the first construct in the checklist. It is important to understand the characteristics of an organisation, as it can have an influence on how an intervention is delivered and its effectiveness. Organisation has been included as a feature in a number of reporting tools such as CONSORT [8] and other checklists [6,12]. Often, this is included in the broader heading of ‘setting’. However, it was important to make the checklist more specific and clear to the researchers. Few changes were made to this construct throughout the development process.

### Location

Another characteristic of a setting is the location. The location construct was considered as important from the beginning of the checklist. In research proposals it is useful to know whether the location is in an area of deprivation, or if it is in an urban or rural location. This will ultimately affect the delivery of the intervention, which may not be generalizable in another location.

### Patient group

Patient group was included from the start of the development of the checklist. Most checklists which are used to report research emphasise the importance of having a good description of the participants. However, the word ‘participant’ can mean a range of groups who take part in a study. For the purpose of the checklist we made this specific to the patient group. Nevertheless, if the intervention is evaluating a workforce intervention, this is covered by the next construct (workforce and staffing). Therefore, the term ‘patient group’ was used as this appeared clearer to what is needed in research applications.

### Workforce and staffing

Workforce and staffing were considered to be important from the outset of the development of the checklist. Elements of this construct have been seen in other checklists such as those used for patient safety [6]; however, the wording has been adapted to suit the needs of this checklist. When originally forming the checklist, workforce and staffing were separate constructs. However, when conducting the retrospective review, the two constructs were merged, as it was difficult to distinguish between the two.

### Intervention

Originally, this construct was called ‘service provided’, as it was felt this would be the best wording to describe the active ingredient. However, there is often confusion about what the active ingredient is, especially when it relies on the context in which it is being delivered. For instance, an intervention such as care closer to home for children with complex conditions might build on some existing community nursing services but with some modification to skill mix or grade mix. As a result, this then overlaps with other constructs, such as ‘workforce and staffing’ and can appear unclear; this became particularly apparent when completing the retrospective review. Therefore, for simplicity, this construct was changed to ‘intervention’ which would be clearer to the researchers.

### Other important contextual information

When initially forming the checklist ‘culture/leadership and costing’ were each considered an essential construct. However, these were difficult to justify and it was not thought appropriate to represent them in a separate single construct, although they were of high importance. Therefore, a construct called ‘Other Important Contextual Information’ was added to cover all other aspects, which would be missed by the other constructs. A number of these elements were considered important, as highlighted in Taylor et al. [6], such as organisational and structural features of provider organisations (size, teaching status, and number of beds) with other facets, which may be more difficult to measure, such as levels of team working, cohesion, and leadership. However, when completing the retrospective review, these were removed from the checklist as it became apparent that it is particularly difficult to describe these in research proposals. Nonetheless, it is important to try and capture these important aspects in a research proposal. Thus, the ‘other important contextual information’ construct was developed to ensure the researchers have considered all characteristics of contextual information in their research proposal, particularly those that affect the generalizability to other sites. One of the main difficulties in health service research is generalising findings from one service to another, given the importance of context and local determinants of practice. Consequently, it was essential that these elements of a research proposal are clear and well described. Examples include any policy initiatives related to the intervention; issues at the study sites around exceptional leadership, for instance championing by nurse director or clinical team and local features such as unusual geographical or service configurations; whether there is participation in an improvement collaborative or Commissioning for Quality and Innovation initiative on particular clinical area; or a relevant pay-for-performance initiative for a clinical pathway.

## Discussion

This paper reports on a small action-orientated mixed methods study which aimed to develop a checklist to be used to aid researchers when writing research proposals to submit to the NIHR HS&DR Programme. There are many constructs which would be useful to a researcher; however, some have changed shape or have been removed at various stages during the development of the checklist. The final six checklist constructs have a blurring between intervention and context, as debated in peer-reviewed literature [5]. The blurring was recognised as a difficulty for researchers, but it has not been possible to keep the two separate. However, as the feedback confirms, having six clear constructs may make applying for research funding more straightforward for less experienced applicants, so the blurring should be less of a concern.

This study’s main strength is that, to our knowledge, this is the first time someone has developed a checklist to aid the development of research proposals in health services and delivery research. There are also several limitations that need to be acknowledged. Firstly, in the retrospective review of research proposals we only included a small selection of research proposals. Future research could look at a different and larger selection of research proposals. Next, only two people conducted the retrospective review. Therefore, there is a possibility that someone with different expertise would have collected different information for the checklist or would have had a different view on the checklist. Lastly, it is important to highlight that only some initial small-scale work was completed with a sample of funded researchers to test usefulness, acceptability, and face validity of the constructs and checklist. Future work could include more rigorous validation of the checklist.

Initial positive feedback from researchers indicated that this checklist may be a useful tool to help address the problem of not being able to fund important research proposals. The work presented in this paper should be considered the first step in a longer-term process of subsequent research. The checklist was developed essentially as an in-house, pragmatic tool for the NIHR HS&DR Programme and further research is needed to strengthen the checklist. It would be useful to test the checklist constructs with a wider sample of stakeholders using techniques such as consensus building or a Delphi model.

## Conclusions

There is a body of literature which discusses intervention, context, and the use of checklists. However, these checklists are not always relevant to complex, staff-based organisational research in healthcare; existing checklists do not have enough focus on areas relevant to complex health service interventions or consider research applications. We have developed a checklist, with a focus on complex health services and delivery interventions and context. Small-scale iterative testing suggested it was acceptable and useful for researchers. Further validation is needed to demonstrate relevance to a wider range of researchers and funding bodies.

## Abbreviations

HS&DR: Health Services and Delivery Research; NHS: National Health Service; NIHR: National Institute for Health Research.

## Competing interests

The authors are employed at the National Evaluation, Trials and Studies Coordinating Centre, which manages the HS&DR Programme on behalf of the NIHR.

## Authors’ contributions

HD led the research and the writing of the paper. All authors contributed to or commented on successive drafts of the paper. The study was conceived and designed by TL. HD and DW conducted the retrospective review. All authors contributed to the steering of the research. All authors read and approved the final manuscript.
